# Therapeutic strategies to slow chronic kidney disease progression

**DOI:** 10.1007/s00467-008-0789-y

**Published:** 2008-05-01

**Authors:** Elke Wühl, Franz Schaefer

**Affiliations:** Division of Pediatric Nephrology, University Hospital Heidelberg for Pediatric and Adolescent Medicine, Im Neuenheimer Feld 151, 69120 Heidelberg, Germany

**Keywords:** Chronic kidney disease, Progression, Children, Prevention, Hypertension, Proteinuria

## Abstract

Childhood chronic kidney disease commonly progresses toward end-stage renal failure, largely independent of the underlying disorder, once a critical impairment of renal function has occurred. Hypertension and proteinuria are the most important independent risk factors for renal disease progression. Therefore, current therapeutic strategies to prevent progression aim at controlling blood pressure and reducing urinary protein excretion. Renin-angiotensin-system (RAS) antagonists preserve kidney function not only by lowering blood pressure but also by their antiproteinuric, antifibrotic, and anti-inflammatory properties. Intensified blood pressure control, probably aiming for a target blood pressure below the 75th percentile, may exert additional renoprotective effects. Other factors contributing in a multifactorial manner to renal disease progression include dyslipidemia, anemia, and disorders of mineral metabolism. Measures to preserve renal function should therefore also comprise the maintenance of hemoglobin, serum lipid, and calcium-phosphorus ion product levels in the normal range.

## Natural course of chronic kidney disease

Progression of chronic kidney disease (CKD) toward end-stage renal failure is common in CKD patients, and once significant impairment of renal function has occurred, it tends to progress irrespectively of the underlying kidney disorder. However, information on the natural course of CKD progression in children is still limited. The prospective, population-based ItalKid registry, including almost 1,200 CKD children with various renal diseases over a 10-year period, reported a prevalence of 23% of patients suffering from severe kidney disease with chronic renal insufficiency (CRI). The incidence of renal replacement therapy was 7.3 per year per 100 patients with CRI, and the risk of developing end-stage renal disease (ESRD) by age 20 was 68% [1]. The decline of renal function was not linear but rather characterized by a sharp decline during puberty and at early postpubertal age. This finding supports the general clinical impression that in many children with renal hypodysplasia, kidney function deteriorates more rapidly around the time of puberty. This notion received further support by a recently published retrospective analysis of 176 children with renal hypodysplasia [2]. The authors postulated that the natural course of chronic renal failure in these patients can be divided into three time periods: an initial period, usually lasting the first 3 years of life characterized by an improving renal function, a subsequent period of stable renal function attained by 50% of patients for a mean of 8 years, and a phase with renal function gradually deteriorating toward ESRD. The latter period started just after infancy in 48% and around puberty in 23%. In 30% of patients, renal function remained stable even beyond puberty.

## Factors affecting renal disease progression

**Fig. 1:**
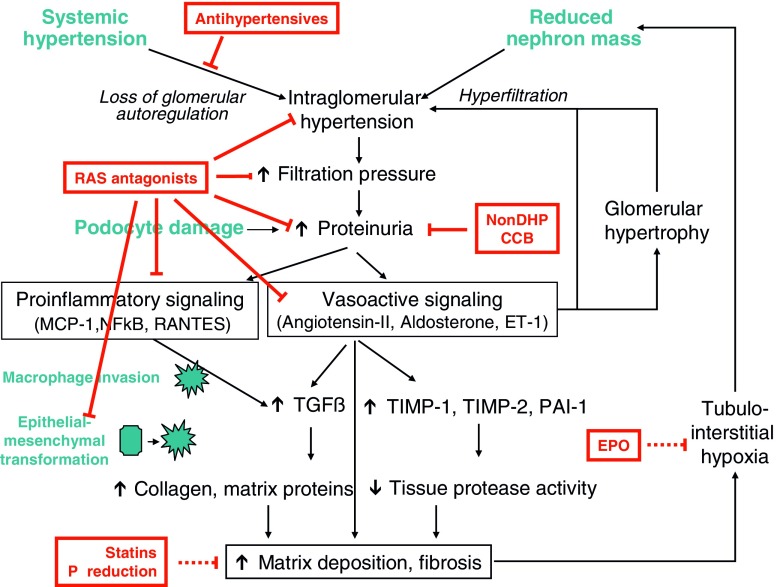
Pathophysiology, consequences, and treatment options of hypertension and proteinuria in chronic kidney disease. *EPO* erythropoietin, *P reduction* serum phosphate reduction, *RAS* renin angiotensin system, *Non-DHP CCP* non dihydropyridine calcium channel blockers, *TGFβ* transforming growth factor β, *TIMP* tissue inhibitors of metalloproteinases, *ET1* endothelin 1, *PAI* plasminogen activator inhibitor, *⊥* inhibitory effect

There is clear evidence from clinical studies that hypertension and proteinuria are key players in the pathophysiology of CKD progression in humans [3–5]. The renin-angiotensin system (RAS) is intrinsically involved in the process, and other potential contributors include genetic background, renal anemia, altered mineral homeostasis, dyslipidemia, chronic inflammation, and oxidative stress as well as general cardiovascular risk factors such as diabetes, smoking, and obesity. As a consequence of the mechanistic insights in renal disease progression obtained by experimental work, several principal renoprotective strategies have emerged in recent years (Fig. 1). These are based mainly on clinical evidence established in adult patients, but growing evidence supports their efficacy also in children. Efficient control of blood pressure and minimization of proteinuria appear as the two most important measures to preserve residual kidney function. Other issues, such as prevention and treatment of renal anemia, uremic dyslipidemia, and disorders of mineral metabolism, have an experimental basis, although their clinical importance is less clear to date. In the following, we review current treatment strategies to slow renal disease progression in childhood CKD.

### Hypertension

Hypertension is an independent risk factor of renal failure progression in adults [3–5]. Whereas the degree of hypertension correlates with the severity of the underlying renal disease, interventional studies have provided evidence that high blood pressure actively contributes to renal failure progression in human CKD. In pediatric nephropathies, renal hypertension is common, although typically less severe than in adult kidney disorders. Hypertension prevalence estimates in children with CKD range from 20% to 80% depending on the degree of renal dysfunction and underlying renal disease [6, 7]. However, even children with CKD stage 2 or renal hypodysplasia may present with significantly elevated blood pressure [8]. The European Study Group for Nutritional Treatment of Chronic Renal Failure in Childhood demonstrated that in CKD children, a systolic blood pressure greater than 120 mmHg was associated with a significantly faster glomerular filtration rate (GFR) decline [9].

Numerous studies in adults have provided proof to the concept that consequent antihypertensive therapy slows down the rate of renal-failure progression [10]. A close linear relationship between the blood pressure level achieved by antihypertensive treatment and the rate of renal failure progression in adult CKD patients has been noted, which appears to persist well into the normal range of blood pressure [11, 12].

The firm evidence of a favorable effect of intensified blood pressure control in patients with CKD has resulted in generally lower target blood pressure recommendations in this patient group. In the most recent guidelines by the Joint National Committee in the US (JNC7) [13] and the Guidelines of the European Hypertension Society [14], 120/80 mmHg has been defined as the upper limit of the ‘optimal’ blood pressure range, particularly when proteinuria is present, and any blood pressure > 130/80 in CKD patients should be actively lowered by therapeutic intervention [15]. These blood pressure targets are equivalent to the 50th to 75th distribution percentile in the general young adult population. It is as yet unknown whether these blood pressure targets hold true for the pediatric population and whether glomerular damage in children correlates with absolute or age-specific relative blood pressure. The Kidney Disease Outcomes Quality Initiative (K/DOQI) guidelines on blood pressure control in CKD children [15] adopted the recommendations of the task force that target blood pressure should be <90th percentile for normal values [16] adjusted for age, gender, and height percentile. Assuming that equivalent blood pressure percentiles should be targeted in children as in adults, the adult recommendations would, for instance, correspond to an acceptable upper blood pressure level of 106/66 mmHg (75th percentile) in an 8-year old child with CKD. The final results of the Effect of Strict Blood Pressure Control and ACE Inhibition on Progression of Chronic Renal Failure in Pediatric Patients (ESCAPE) trial, to become available in mid-2008, will provide pediatric evidence as to whether intensified blood pressure control (targeting to below the 50th percentile of 24-h mean arterial pressure) will confer a renoprotective advantage over a more conventional target (50th to 95th percentile) [17]. The integrity of the normal diurnal blood pressure pattern may play a significant role in renal failure progression in addition to and independent of the absolute blood pressure level. Nondipping, a well known independent cardiovascular risk factor and common characteristic of renoparenchymal hypertension, is associated with more rapid progression of renal failure in adult CKD patients [18, 19], and nondipping is believed to reflect sympathetic hyperactivation in CKD.

### Proteinuria

Population-based studies in healthy individuals have demonstrated that proteinuria is a powerful independent risk factor for ESRD and overall mortality [20–22]. Proteinuria is certainly predictive of the renal prognosis in adults with diabetic and nondiabetic kidney disorders [23–25]. Urinary protein excretion was the only baseline variable correlated with GFR decline and progression to ESRD in the Ramipril Efficacy in Nephropathy (REIN) trial [26]. However, the spectrum of underlying renal disorders in children differs markedly from adults. In the pediatric CKD population, congenital renal hypodysplasia with or without urinary tract abnormalities is the leading underlying renal disorder, affecting more than 60% of children. The European Study Group for Nutritional Treatment of Chronic Renal Failure in Childhood first demonstrated in 200 children with CKD stage 3–4 that proteinuria and hypertension are major independent determinants of GFR decline in pediatric nephropathies [9]. The ItalKid Project confirmed that proteinuria predicts renal disease progression in children with renal hypodysplasia [27]. In addition, there is evidence from the ESCAPE trial that residual urinary protein excretion during angiotensin-converting enzyme (ACE) inhibition is quantitatively associated with renal failure progression [28]. Even in children with normal kidney function, persistent proteinuria in the nephrotic range is a risk factor for progressive renal injury, and early detection and treatment of proteinuria is essential [29]. In nonproteinuric children with CRI of nonglomerular origin, the level of protein excretion does not appear to play a major role in CKD progression, which seems to be best predicted by rapid somatic growth, age, and blood pressure [30].

In line with evidence from animal models, multiple clinical studies have confirmed that proteinuria is not only a marker but also an important mechanism of CKD progression. Reduction of proteinuria is associated with a slowing of GFR loss in the long term [25, 31–33]. In the Modification of Diet in Renal Disease (MDRD) trial, for each 1 g/d reduction in proteinuria observed within 4 months of antiproteinuric treatment (i.e. blood pressure reduction and dietary interventions), the subsequent GFR decline was slowed by about 1 ml/min per 1.73 m^2^ per year [25]. In the REIN study, reduction of proteinuria at 3 months of ACE inhibitor therapy by 1 g/d resulted in slowing down GFR decline by 2 ml/min per year [34]. This degree of proteinuria reduction appears to be associated with the maximal renoprotective effect [35, 36]. Hence, the goal of any antiproteinuric treatment is to reduce proteinuria as much as possible, ideally to <300 mg/m^2^/day.

### Dyslipidemia

Epidemiological studies suggest that dyslipidemia is an independent risk factor not only for cardiovascular disease but also for progressive chronic renal failure [37]. The dyslipidemic pattern differs between the major renal disease entities [38], and the degree of dyslipidemia parallels the degree of renal function impairment. In animal models, hypercholesterolemia clearly accelerates the rate of progression of kidney disease [39]. A high-fat diet causes macrophage infiltration and foam-cell formation in rats, leading to glomerulosclerosis [40]. Dyslipidemia may damage glomerular capillary endothelial and mesangial cells as well as podocytes. Macrophages are the major cell types expressing scavenger receptors; however, mesangial cells, as well, express receptors for low-density lipoprotein (LDL) and oxidized LDL, which upon activation induce mesangial cell proliferation, increase mesangial matrix deposition, and enhance production of chemokines, cytokines, or growth factors and increase oxidative stress.

A relationship between serum cholesterol levels and GFR decline was shown in adult patients with type 1 diabetes and overt nephropathy [41]; patients with a total cholesterol level >7 mmol/L showed an at least three times faster decline in GFR than subjects with lower cholesterol levels. For the general adult population, the Arteriosclerosis Risk in Communities (ARIC) study demonstrated that elevated triglycerides and low high-density lipoprotein (HDL) cholesterol but not LDL cholesterol were associated with an increased risk of renal dysfunction [37]. In a cohort of more than 11,000 middle-aged adults with normal kidney function, hypertriglyceridemia was associated with a 1.68 times increased risk of a 0.4 mg/dl increase in serum creatinine within the 3-year observation period [37].

There are also observations that insulin resistance syndrome may underlie or mediate the association between lipids and loss of renal function. In humans, a strong relationship between metabolic syndrome and the risk for chronic renal disease and microalbuminuria was found in a large nondiabetic general population [42].

### Anemia

There is increasing evidence that anemia is an independent risk factor for progression of chronic renal failure. Anemia is a surrogate marker for tissue hypoxia that might perpetuate preexisting renal tissue damage. In patients with reduced nephron number, hypoxia of tubular cells is favored by an increase of oxygen consumption by tubular cells of the remaining nephrons, a decrease in the number of interstitial capillaries [43], and an accumulation of extracellular matrix between interstitial capillaries and tubular cells, which hampers oxygen diffusion. Hypoxia appears to have at least three consequences: It stimulates production of profibrotic molecules such as transforming growth factor (TGF)-β or endothelin-1 by tubular cells, synthesis of extracellular matrix [44], and increased oxygen consumption, which also enhances production of reactive oxygen species (ROS) that may play an additional role in CKD progression.

The renoprotective effect of erythropoietin (EPO) in CKD might be partially related to an attenuation of interstitial fibrosis and tubuloepithelial cell loss by improved oxygen supply and reduced oxidative stress via correction of anemia. In addition, EPO might exert direct protective effects on tubular cells and might help maintain integrity of the interstitial capillary network and stimulate regenerative progenitor cells [45]. The combination of antiapoptotic effects of rhuEPO in renal tissue and stimulation of regenerative progenitor cells may play a role in organ protection.

### Oxidative stress

Oxidative stress is defined as an imbalance between ROS and endogenous levels of antioxidant substances. High oxidative stress and low availability of the substrate of nitric oxide (NO) synthase, L-arginine, and an accumulation of endogenous NO inhibitors such as asymmetric dimethylarginine (ADMA) may induce endothelial dysfunction. Several studies reported on increased oxidative stress in CKD patients. This increase appears to correlate with the extent of deterioration of renal function [46]. Increased oxidative stress contributes to the release of proinflammatory and profibrotic molecules and thereby directly enhances the production of extracellular matrix by fibroblastic cells. This may lead to accelerated progression of CKD, hypertension, and cardiovascular complications, and it was suggested that increased oxidative stress in CKD patients may be both a cause and an effect of renal injury. Anemia, hypercholesterolemia, and chronic inflammation are conditions known to further promote oxidative stress.

### Nutrition

Nutrition has been considered an important instrument for slowing down renal disease progression in individuals with impaired renal function. In animals, high protein diet results in renal scarring, whereas restriction of dietary protein diminishes or even prevents progressive renal damage [47]. In end-stage renal failure, uremic symptoms can be often diminished and renal replacement therapy postponed by the restriction of dietary protein intake. This led to the hypothesis that restriction of protein intake might also slow down the progression of chronic renal failure in patients with CKD stages 2–4.

### Disorders of calcium-phosphate metabolism

Disorders of the calcium-phosphate metabolism are additional risk factors for renal disease progression. On one hand, renal insufficiency causes disturbances of the calcium-phosphate homoeostasis and alters serum lipid profiles. On the other hand, the resulting vasculopathy and hypertension promote progression of chronic renal failure toward end-stage renal disease. Several factors related to disturbed calcium-phosphorus metabolism, such as hyperphosphatemia, hyperparathyroidism, lack of active vitamin D, and possibly the phosphaturic hormone FGF23, may be considered to be—at least to a minor extent—involved in the progression of renal dysfunction [48].

## Treatment strategies and their impact on renal disease progression

Several antihypertensive and antiproteinuric therapies have proven effective. Blood pressure control per se has a proteinuria-lowering effect, as demonstrated by three large trials: the MDRD study [25], the Appropriate Blood Pressure Control in Diabetes (ABCD) study [49], and the African American Study of Kidney Disease and Hypertension (AASK) [32]. A low blood pressure goal, i.e. <125/75 mmHg in adults, either reduced proteinuria absolutely by 50% [25] or prevented the two- to threefold increase in proteinuria observed in patients with the more conventional blood pressure goal of 140/90 mmHg [49]. A low blood pressure goal appears to be very well tolerated by the vast majority of patients and in terms of cardiovascular outcomes; the “J curve” phenomenon (a slight increase of cardiovascular events in patients achieving a very low blood pressure level) seems to be confined to aged patients with advanced atherosclerosis.

The goal of any antiproteinuric treatment is to reduce proteinuria as much as possible, ideally to <300 mg/m^2^/day. This degree of proteinuria reduction appears to be associated with the maximal renoprotective effect [35, 36]. Whereas the different classes of antihypertensive agents are comparable with respect to their blood pressure-lowering efficacy, they differ markedly regarding their effects on proteinuria and CKD progression [32, 35, 50, 51].

### Blockade of the renin-angiotensin system

Antagonists of the RAS, such as ACE inhibitors and, more recently, angiotensin II type I receptor blockers (ARB) have become pharmacotherapeutics of first choice in adults [15] as well as children with CKD by virtue of their pharmacological properties. They significantly reduce blood pressure as well as urinary protein excretion and have an excellent safety profile, which is almost indistinguishable from placebo. In adults with essential hypertension, treatment with RAS antagonists has been associated with the best quality of life among all antihypertensive agents.

RAS antagonists suppress the local angiotensin II tone (ACE inhibitor) or action (ARB). This results in a reduction of intraglomerular pressure and proteinuria, diminished local release of cytokines and chemokines, and alleviated activation of inflammatory pathways, with consequently attenuated glomerular hypertrophy and sclerosis, tubulointerstitial inflammation, and fibrosis [8], as well as in a normalized central nervous sympathetic tone by reduced renal afferent nerve stimulation. In addition, oxidative stress is reduced independently of the blood-pressure-lowering effect [52].

In adults with diabetic or nondiabetic kidney disease, several randomized trials demonstrate a more effective reduction of proteinuria, usually by 30–40%, by ACE inhibitors compared with placebo and/or other antihypertensive agents [35]. This is associated with a significantly reduced rate of renal failure progression in the long term [31, 35, 53–61].

Very similar results were obtained in randomized studies comparing ARBs with placebo or conventional antihypertensive agents in diabetic nephropathy [51, 62, 63]. It has been reasoned that ACE inhibitors might have a specific renoprotective advantage by inducing accumulation of vasodilatory and antifibrotic bradykinins; however, the course of GFR was similar in two clinical trials comparing ACE inhibitors and ARB therapy [64, 65]. The size of the advantage of RAS antagonists over other antihypertensive agents is still under debate [66]. The risk of doubling serum creatinine or attaining ESRD is typically reduced by 30–40%, but the superiority of RAS antagonists is related to the prevailing degree of proteinuria [35, 36]. In adults, ACE inhibitors are believed to provide better renoprotection than other antihypertensive agents in patients with proteinuria exceeding 500 mg/day.

However, there is some evidence that previous studies may not have used sufficiently high ACE inhibitor doses to achieve effective RAS suppression at the kidney tissue level and obtain a maximal renoprotective effect. Furthermore, at least a subset of patients appears to develop partial secondary resistance to ACE inhibition (aldosterone escape by compensatory upregulation of ACE-independent angiotensin II production) [67–69]. It is currently an open issue whether such patients would benefit from the primary use of ARBs alone or in combination with ACE inhibitors.

Whereas the maximal antiproteinuric and renoprotective effects of ACE inhibitors and ARBs seem to occur at doses that are supramaximal with respect to maximal antihypertensive action, regulatory authority approval is usually available only for the indication of hypertension in the respective dose range. Therefore, it is generally recommended to administer these drugs, after confirming tolerability in a short run-in period, at their highest approved doses [32, 70].

Limited information is available regarding the efficacy of RAS antagonists for renoprotection in children with CKD. Small uncontrolled studies showed stable renal function in children with sequelae of hemolytic uremic syndrome during long-term ACE inhibitor treatment [71], stable GFR during 2.5 years of losartan treatment in children with proteinuric CKD [72], and attenuated histopathological progression in children with IgA nephropathy receiving combined RAS blockade [73]. Data from the ItalKid Study did not show a significant modification of CKD progression by ACE inhibitor treatment in children with hypodysplastic kidney disease [74] compared with matched untreated subjects. However, the overall CKD progression rate in the total cohort was very slow (< –2 ml/min per 1.73 m^2^ per year), thereby making the detection of significant differences (ACE inhibitors -1.08 vs. non-ACE inhibitors 1.80; not significant) difficult. In addition, no information was available with respect to the types and dosages of ACE inhibitors used and the prevailing degree of proteinuria.

The ESCAPE trial demonstrated efficient blood pressure and proteinuria reduction by ramipril in almost 400 children with CKD [17]. However, an interim analysis of the 3-year results revealed a gradual rebound of proteinuria after the second treatment year. This effect was dissociated from a persistently good blood pressure control and may limit the long-term renoprotective efficacy of ACE inhibitor monotherapy in pediatric chronic kidney disorders [28].

Aldosterone antagonists also lower blood pressure by RAS suppression. Whereas the use of spironolactone is limited by endocrine side effects, the new aldosterone antagonist, eplerenone, has minimal affinity for progesterone and androgen receptors. Apart from the risk of hyperkalemia, reported side effects are similar to placebo [75]. Combined therapy of eplerenone and an ACE inhibitor increases patient survival in adults with congestive heart failure [76]. However, combination therapy appears limited in CKD patients due to the potentiated risk of hyperkalemia [77, 78].

Aliskiren, a renin-antagonist, blocking the conversion from angiotensinogen to angiotensin I, has been shown to effectively lower blood pressure in animals and humans. The effect on blood pressure is comparable with that of ARBs, and combination therapy of aliskiren and valsartan at maximum recommended doses provided significantly greater reductions in blood pressure than did monotherapy, with a tolerability profile similar to that of aliskiren or valsartan alone [79]. However, there are no data on the effect of aliskiren on renal disease progression in adults nor on its applicability in children available to date.

### Calcium-channel blockers

Calcium-channel blockers (CCBs) are safe and can achieve blood pressure goals in patients with CKD. However, CCBs of the dihydropyridine type (amlodipine, nifedipine) fail to reduce progression of chronic renal failure and may even increase proteinuria and promote more rapid CKD progression [33]. Therefore, dihydropyridine CCBs may be acceptable as first-line antihypertensive monotherapy only in nonproteinuric patients and should be avoided unless in combination with RAS antagonists to improve blood pressure control in proteinuric patients [70]. In contrast, nondihydropyridine CCB (diltiazem, verapamil) may have some antiproteinuric effect and may be therefore renoprotective [33]. However, data are not conclusive. An antiproteinuric effect was not observed in type 2 diabetes [80], and amlodipine exerted a renoprotective effect comparable with ACE inhibitors in one study [81].

### Beta-blockers

CKD is often a state of overactivation of the sympathetic nervous system, and antiadrenergic drugs play an important role in its management. Beta-blockers are effective in lowering blood pressure in CKD patients by blockade of postsynaptic beta receptors, resulting—among others effects—in a reduction of pulse rate, cardiac output, afterload, and renal renin release. Metoprolol and atenolol were the first antihypertensive agents for which beneficial effects on the decline of renal function in CKD patients were demonstrated [54]. In the AASK trial, the beta-blocker metoprolol had an antiproteinuric effect almost comparable with ramipril, in marked contrast to amlodipine [32]. The antiproteinuric action may be due to sympathicoplegic effects. Newer beta-blockers, such as carvedilol, have even improved antiproteinuric effects compared with atenolol [82, 83].

### Combination therapy

Because hypertension is a multifactorial disorder, monotherapy is often not effective in lowering blood pressure or reducing proteinuria to the target range. Treatment with a single antihypertensive agent usually controls blood pressure in less than half of the patients. According to the JNC7 guidelines, subjects with blood pressure >20/10 mmHg above the normal range (i.e. >160/100 mmHg in adults) should be started on combination drug therapy [13]. In CKD patients, RAS antagonists are most commonly combined with a diuretic or with a CCB, whereas their combination with a beta-blocker usually does not exert an additive effect on blood pressure control. Fixed-dose combination preparations are becoming increasingly popular in antihypertensive therapy and may help maximize treatment adherence and efficacy.

Combined RAS blockade using ACE inhibitors and ARB concomitantly has only a minor effect on blood pressure (3–4 mmHg vs. monotherapy) but increases the antiproteinuric effect of ACE inhibitors or ARB monotherapy by 30–40% [64, 84–86]. The prospective randomized Combination Treatment of Angiotensin II Receptor Blocker and Angiotensin-Converting-Enzyme Inhibitor in Non-diabetic Renal Disease (COOPERATE) trial, performed in adults with nondiabetic nephropathies, suggested that combination therapy may also provide better long-term renoprotection [64]. However, in most ACE inhibitor/ARB combination studies, it remained unclear whether maximally efficient single-drug doses were used, a formal prerequisite to demonstrate true synergism of the two drug classes. The few published studies assessed the effects of single-drug dose escalation followed by combined administration of an ACE inhibitor and an ARB at maximally effective single doses found synergistic antiproteinuric effects of combined treatment [87]. Otherwise, a recent study demonstrated additional proteinuria reduction by escalating candesartan exposure to an ultrahigh dose [88]. Notably, raising the dose from 16 to 32 mg daily had no effect on proteinuria, whereas a further increase from 32 to 64 mg was highly effective, suggesting that the dose–response relationship may be nonlinear. Hence, the issue of whether ACE inhibitor and ARB combination therapies have a synergistic renoprotective potential remains an exciting field of clinical research.

### Restoration of blood pressure day–night rhythm

In view of the fact that nondipping of nocturnal blood pressure is an independent risk factor for CKD progression, effects of the timing of application of antihypertensive drugs may be an issue of interest. Even using agents with a long half-time and recommended administration on a once-daily basis, evening administration lowers nighttime blood pressure more effectively, increasing the day–night ratio and partially restoring the physiological nocturnal dipping pattern. However, these effects seem to differ for individual antihypertensive drug classes. Whereas bedtime administration of CCBs and ACE inhibitors tends to restore the dipping pattern, evening dosing of beta-blockers has no effect on the circadian blood pressure rhythm [89]. In a substudy of the Heart Outcomes Prevention Evaluation (HOPE) trial, adult patients were evaluated by ambulatory blood pressure monitoring (ABPM) after evening administration of the ACE inhibitor ramipril. A more marked blood pressure reduction during nighttime was observed, compatible with the notion that the beneficial effects of ramipril on cardiovascular morbidity and mortality in the HOPE study were related to the 8% increase in the day–night ratio of blood pressure obtained with evening dosing [90]. Although this association appears firm, it is as yet unclear whether pharmacological restoration of the dipping pattern will result in any long-term clinical benefit for cardiovascular health in general and for renal function preservation in CKD. However, it is of note that the antiproteinuric efficacy of the ARB valsartan was found correlated with the increase in blood pressure day–night ratio induced by evening dosing [91].

### Treatment of dyslipidemia

General measures to prevent dyslipidemia in CKD patients include prevention or treatment of malnutrition and correction of metabolic acidosis, hyperparathyroidism, and anemia, all of which may contribute to dyslipidemia [92–94]. In addition, referring to evidence from the general population, therapeutic life-style modification is recommended for adults and children with CKD-related dyslipidemia [95]. However, the lipid-lowering effect of life-style modifications in CKD patients is usually not impressive. Lipid-lowering medical treatment is commonly prescribed in adults with CKD based on the evident benefit of this approach for primary and secondary prevention of cardiovascular disease in the general adult population. Statin therapy is effective in reducing cardiovascular morbidity and mortality in adults with moderate to severe CKD although not in patients with ESRD [96, 97].

With respect to renoprotection, experimental evidence suggests that statins may retard renal disease progression not only by their lipid-lowering but also by lipid-independent pleiotropic effects. Statins inhibit signaling molecules at several points in inflammatory pathways. Anti-inflammatory effects, reduction of oxidative stress, and improved endothelial function are thought to be partially responsible both for CVD risk reduction and improved renal function [98]. Furthermore, there is also evidence for synergistic effects of statins and RAS inhibitors on the prevention of renal disease progression [99]. However, a recent meta-analysis of published clinical trials concluded that the intrinsic antiproteinuric and renoprotective effects of statins, albeit significant, are quantitatively small [100]. To date, no studies have evaluated the usefulness of statins in children with progressive nephropathies.

### Erythropoietin treatment

In rats undergoing acute ischemic renal injury, pretreatment with recombinant human erythropoietin (rhuEPO) reduces renal dysfunction and morphological damage. This effect appears to be mainly mediated by a reduction of apoptotic cell death [101]. Darbepoetin, a long-acting EPO analog, ameliorated podocyte injury and decreased proteinuria by maintenance of the podocyte actin cytoskeleton and nephrin expression in puromycin-induced nephrotic rats [102]. Even more interesting than treatment of acute renal failure may be tissue protection in chronic renal failure. In a recently published clinical trial, early initiation of rhuEPO therapy in patients with CKD and mild to moderate anemia significantly slowed down the progression of renal disease and delayed the need for renal replacement therapy [103]. However, other data in patients with more advanced CKD and high-dose rhuEPO treatment revealed no beneficial effect on renal survival [104]. The role of EPO in pediatric CKD progression has not been defined yet.

### Nutrition and vitamin D supplementation

For decades, low-protein diets have been prescribed for preventing CKD progression. However, the effects of these diets on CKD progression and delay of ESRD are still inconclusive. One of the largest trials, the MDRD trial, could not prove efficacy of a low-protein diet on progression in nondiabetic kidney disease [105], whereas a recent Cochrane Review [106] found a risk reduction of renal death in patients with protein restriction. Thus, the progression rate was not significantly influenced by protein restriction, whereas renal replacement therapy could be postponed. In children, reducing protein intake to the maximal acceptable lower limit was ineffective to slow down renal disease progression [9, 107]. Further reductions may be effective but not acceptable. Furthermore, therapeutic strategies of protein reduction in children may be conflicting, since a low-protein diet bears the risk of low calorie intake, whereas a high calorie intake is needed for optimal growth. Therefore, at present, it seems not to be justified to prescribe low-protein diets to children early in the course of chronic renal failure.

Some studies in adult CKD patients suggest that dietary phosphorus restriction may stabilize kidney function [108]. However, conclusions in this regard could not be drawn from studies in children [109]. A high calcium–phosphorus product may be detrimental to renal survival by aggravating intrarenal vasculopathy as well as by causing tubulointerstitial calcifications, which may stimulate tubulointerstitial inflammation and fibrosis. In view of these pathophysiological associations, it is currently discussed whether calcium-free phosphate binders may have some renoprotective potential in patients with CKD. Sevelamer may prove beneficial beyond phosphate lowering due to its pleiotropic effects, which include lipid-lowering and anti-inflammatory properties. Treatment with nonhypercalcemic doses of active vitamin D attenuates renal failure progression in chronically uremic rats. This effect may be brought about by the immune modulatory and antifibrotic properties of vitamin D. In addition, a negative endocrine regulation of the RAS through 1,25-Dihydroxyvitamin D_3_ has been reported [110]. In humans, an antiproteinuric effect of oral paricalcitol was demonstrated in adult CKD patients [111]. These exciting experimental and early clinical findings provide an additional rationale beyond mineral metabolism for close monitoring and early intervention to maintain mineral, vitamin D, and PTH homeostasis in CKD [109].

## Conclusion

In conclusion, hypertension and proteinuria are key players in renal disease progression. Therapeutic strategies to prevent progression should comprise blood pressure control and lowering of proteinuria. RAS antagonists preserve kidney function, not only by lowering blood pressure but also through antiproteinuric and antifibrotic properties. Other factors contributing to renal disease progression in a multifactorial manner include anemia, dyslipidemia, and disorders of mineral metabolism, and measures to preserve renal function should therefore also comprise the maintenance of hemoglobin and serum lipid and calcium-phosphorus ion product levels in the normal range.

## Questions

(Answers appear following the reference list.)

1. Progression of chronic renal failure toward ESRD is common in children with CKD and:
  - Declines linearly
  - Nonlinearly and often characterized by a sharp decline in renal function during puberty
  - Inevitably
  - Strongly, depending upon the underlying renal disease
  - Results in ESRD in less than 50% of patients at age 20 years
2. The most important factors influencing renal disease progression are:
  - Age at onset of chronic renal failure, gender, and underlying renal disease
  - Residual renal function and blood pressure
  - Blood pressure and proteinuria
  - Rapid somatic growth during puberty, and age
3. The different antihypertensive drug classes are comparable regarding:
  - Antihypertensive efficacy
  - Antiproteinuric efficacy
  - Side effects and safety profile
  - Effect on CKD progression
4. The antihypertensive and antiproteinuric effects of RAS antagonists are:
  - Strictly dose dependent
  - Basically mediated by reduction of systemic hypertension
  - Mediated by bradykinin release
  - Also mediated by antifibrotic and vasodilatory effects
  - Due to restoration of the often disturbed day–night blood pressure pattern (dipping) in CKD patients
5. Angiotensin receptor antagonists in children:
  - Are approved for the indication proteinuria and hypertension
  - Do not exert additional antiproteinuric effect when combined with ACE inhibitors
  - Should be given at the highest approved dose for maximal antiproteinuric effect
  - Should not be combined with ACE inhibitors
  - Have the same side effect profile as ACE inhibitors
6. Decline of GFR:
  - Is independent of serum cholesterol level
  - Can be reversed by dietary protein restriction
  - Can be reversed by the use of statins in younger children
  - May be accelerated by prescription of erythropoietin
  - May be retarded by the lipid-independent pleiotropic anti-inflammatory effects of statins
